# Effective pollination of *Aeschynanthus acuminatus* (Gesneriaceae) by generalist passerines, in sunbird-absent East Asia

**DOI:** 10.1038/s41598-019-53035-2

**Published:** 2019-11-26

**Authors:** Kai-Hsiu Chen, Jing-Yi Lu, Chun-Neng Wang

**Affiliations:** 1 Department of Life Science, National Taiwan University, Taipei, 10617 Taiwan; 2 Institute of Ecology and Evolutionary Biology, National Taiwan University, Taipei, 10617 Taiwan; 3 Present Address: Department of Ecology and Evolution, University of Lausanne, Lausanne, 1015 Switzerland; 4 Present Address: Committee on Evolutionary Biology, University of Chicago, Chicago, IL 60615 US

**Keywords:** Evolutionary ecology, Plant ecology, Plant evolution, Pollination

## Abstract

*Aeschynanthus* (Gesneriaceae), a genus comprising approximately 160 species in subtropical Southeast Asia, has red, tubular flowers, typical of a sunbird pollination syndrome. *A. acuminatus*, the species that is distributed extending to the northern edge of the genus, where the specialized nectarivorous sunbirds are absent, possesses reddish-green flowers and a wide-open corolla tube, flowering time shifts from summer to winter and the species achieves high fruiting success. This atypical flower led us to investigate the pollination biology of this species. Three species of generalist passerines, Grey-cheeked Fulvetta (*Alcippe morrisonia*, Sylviidae), White-eared Sibia (*Heterophasia auricularis*, Leiothrichidae) and Taiwan Yuhina (*Yuhina brunneiceps*, Zosteropidae), were recorded visiting *A. acuminatus* flowers. Pollination effectiveness was quantified via conspecific pollen presence on stigmas and natural fruit set. The significantly high natural fruit set (60%) and conspecific pollen transfer rate (94%) indicate high reproductive success facilitated by the accurate pollen placement on the birds. The existence of copious (61 µL) and highly diluted (7%) hexose-dominant nectar, together with a major reflectance peak of corolla lobe in the long-wavelength red color spectrum, is consistent with the pollination syndrome of generalist passerines. The high pollination effectiveness of *A. acuminatus* due to the recruitment of generalist passerines as pollinators, and the specializations of floral traits to match generalist bird pollination, appear crucial in the successful colonization on islands such as Taiwan that lack specialized bird pollinators.

## Introduction

Selection pressure imposed on angiosperms by pollinators greatly contributes to the diversification of flowers^1,2^. Species that are specialized for bird pollination, the “ornithophilous pollination syndrome”, generally feature several specific floral characteristics, such as red, narrow corolla tubes; lack of a landing platform; copious, relatively dilute nectar; and lack of odor^3–5^. Theories explaining each component of these syndromes have been developed through numerous experimental and observational studies. Red color may attract bird pollinators or to deter unwanted floral visitors such as bee^3,6–9^. The narrow corolla tubes help to deposit pollens onto exact position of bird bodies. The corolla lacks the landing platform so that birds can only hover or perch from adjacent stems or branches, which also restricts the bird visiting flowers in certain direction for effective pollination. Birds require significant rewards so that flowers must produce copious nectars^10^. Bird-pollinated flowers usually produce relatively dilute nectars (ca. 20–25% w/w) compared with that is 36% for the nectars of bee flowers^11,12^. The reasons why bird-pollinated flowers produce dilute nectar include, for example, reduced viscosity allows efficient extraction of nectars by bird and water is need for birds’ dietary, or discourage bee visiting^13^.

However, the degree of evolutionary specialization between nectar-feeding birds and their food plants varies greatly among regions. Bird pollination is common in warm tropical and subtropical regions across Asia, Africa and America as nectar supply for nectarivorous birds such as sunbirds and hummingbirds are sufficient all year-round. On the other hand, effective nectarivorous bird pollinations are almost entirely absent in temperate region of Europe and in Asia north of the Himalayas^14–16^. In temperate Asia, the nectarivorous bird specialist (sunbird) absence region, there were growing numbers of reports that opportunistic nectar-feeding birds (generalist passerines) act as alternative pollinators especially for winter flowering plants. The nectar of these winter flowering plants has been found as an important food resource for opportunist birds when there is a shortage of fruits and insects in winter^10,17^. Many winter flowering plants in temperate Australia evolved bird pollination syndrome because birds such as honeyeaters, lorikeets and passerines are more reliable pollinators than insects when the climate and flowering season are unpredictable^10,18–20^.

It is not clear why certain plants in temperate Asia and many plants in cool-temperate Australia switched to winter flowering for evolving the strong interaction from bird pollination. It is possible, however, that birds may provide a better service than insect for greater success of seed set and increase the chance of outcrossing as they fly farer between plants^10,15^. In temperate Asia, generalist passerines have been demonstrated the most effective pollinators for winter flowering plants such as *Brandisia hancei, Camellia* spp., *Eriobotrya japonica and Rhododendron* spp^14,17,21–23^. These opportunistic nectar-feeding birds had been acted as seasonally specialist birds for these winter flowering plants to achieve reproductive success, because in winter these flowers were visited almost exclusively by these generalist passerines despite their omnivorous diet over the remainder of the year^14,24^. For example, in sunbird absence East Asia, recent two studies have identified a generalist passerine, the Japanese white-eye (*Zosterops japonicus*), constitute the majority of the pollinator assemblage for the winter flowering loquat (*Eriobotrya japonica*) in central China, and the common camellia (*Camellia japonica*) in Japan^14,17^. To compensate for the decreased activity of insect pollinators in late winter, *E. japonica* reward passerines with relatively large volumes of dilute nectar and the fruit set of *E. japonica* and *C. japonica* strongly relies on adequate visitation by white-eyes^14,21^. In comparison, the reproductive success of Chinese *Camellia petelotii* also relies heavily on bird visiting but its pollinator is nectarivorous specialist sunbird *Aethopyga siparaja*. The resulting fruits set of passerine-pollinated *C. japonica* is at a similar level (20–30%) to that of sunbird-pollinated *C. petelotii*, suggesting generalist passerine may act as an effective pollinator in the sunbird-absent regions of East Asia^14,23^.

**Figure 1:**
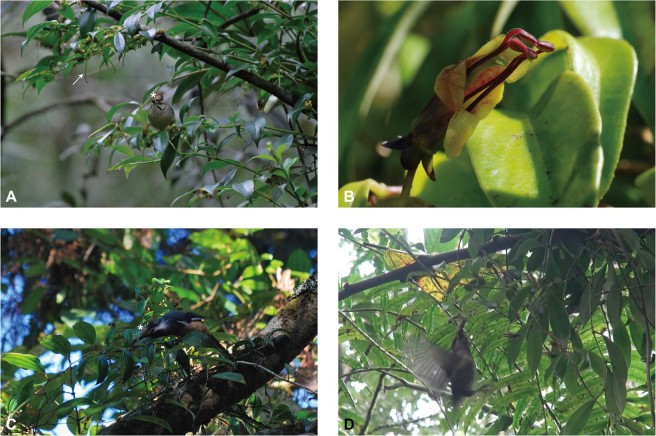
Habitat, flowers, and pollinators of *Aeschynanthus acuminatus*. (**A**) Flowering branches climbing as an epiphyte, with a perching Taiwan Yuhina (*Yuhina brunneiceps*). The white arrow points to an example of an elongated stigma. In this photograph, taken at the end of the flowering season (March) at MYY, many successfully pollinated flowers can be observed starting to develop into fruits. The white asterisk marks the clearly visible yellow spot of pollen on the bird’s head following flower visitation. (**B**) Flower at male stage with stamens exerted and anthers coherent in pairs at apex. (**C**) White-eared sibia (*Heterophasia auricularis*) visited the flowers for nectar by perching at XT. The white asterisk also demonstrates the yellow spot of pollen on the bird’s head. (**D**) Frames captured from the video recordings at QSB show Taiwan Yuhina’s visiting by hovering.

The southeast Asian species of the genus *Aeschynanthus* (Gesneriaceae) have long been considered to be pollinated by sunbirds and spider hunters, although there exists only few observations and little evidence of this^25–27^. Although the diversity center of *Aeschynanthus* is located in Southeast Asia, a few species have successfully spread to East Asia. In particular, *Aeschynanthus acuminatus* Wall. ex A.DC., a plant species that grows outside of the distribution of sunbirds, is of interest. *A. acuminatus* grows as a liana in temperate broadleaf forests (Fig. 1A), and its distribution ranges from mountains of north India, Indo-China, and south China to island Taiwan. It has one of the widest geographical distributions of the 160 *Aeschynanthus* species, and its floral characteristics are quite distinct from the other typical *Aeschynanthus* species. Not like other *Aeschynanthus* species whose flowers are usually equipped with long reddish corolla tubes and flowering in summer, flower of *A*. *acuminatus* is yellowish-green rather than showy red, and it blooms only in the winter, from October to March^27^. Its corolla tube is also relatively shorter and wider than that of related species (Fig. 1B). According to the reconstructed phylogeny and biogeography^28^, *Aeschynanthus* originated in Southeast Asia followed by a vicariance event into mainland Southeast Asia (Clade I, India-Indochina clade) and Malasia (Clade II, Philippine and New Guinea). The topology from Denduangboripant, *et al*.^28^ suggested *A. acuminatus* falls into the Indochina Clade I, and none of its close relatives has similar floral morphology and color. Thus, the distinct traits of *A. acuminatus* are likely to be derived in this genus.

Comparing to continent, insular flora is generally characterized by simpler pollination networks due to the lacking of diverse pollinator fauna^29^. After colonization to islands, plant species are likely to evolve new and novel flower-pollinator interactions^19,30^. *A. acuminatus* is flowering in insect deficient winter in East Asia island such as Taiwan, where the bird pollination is generally lacking. However, based on specimen records and our field observation, the fruit set of *A. acuminatus* is abundant, and individuals are spreading throughout the broadleaf forest, indicating that the species can adapt to various habitats and achieve high reproductive success. Therefore, the extended distribution of *A. acuminatus* to Taiwan, outside the distribution range of sunbirds, the putative pollinators of the plant genus, raises a question of whether *A. acuminatus* has adopted different bird pollinators or shifted to other pollinator classes to achieve high reproductive success.

In this study, we aim to: (a) reveal the pollinators of *A. acuminatus* and evaluate the pollination effectiveness; (b) quantify major floral traits considered characteristic of the pollination system in *A. acuminatus*. We tested the following hypotheses: (1) In the absence of sunbirds, *A. acuminatus* exhibits a pollinator shift, adopting different pollinators different from other bird-pollinating *Aeschynanthus* species. (2) If opportunistic nectar-feeding birds such as generalist passerines act as alternative pollinators for winter-flowering *A. acuminatus*, this pollination may not be effective. (3) If the pollination effectiveness is high, the observed changes of pollination syndromes, specifically the floral color, floral shapes and nectar properties, may match the alternative pollinators to facilitate reproductive success.

## Results

### Pollinator observations

All the visiting passerines were observed in the morning, between 10:00 and 12:00. The flocks of passerines active in the understory usually numbered about 20 individuals, of the following species: Taiwan Yuhina (*Y. brunneiceps*), grey-cheeked fulvetta (*Alcippe morrisonia*), and White-eared Sibia (*Heterophasia auricularis*). Among the three, the fulvetta was observed at QSB and MYY and the Yuhina was recorded at all three study sites, usually in large flocks; while the Sibia was commonly seen on *A. acuminatus* in only one to two individuals at XT. These generalists were recorded as effective pollinators, probing *A. acuminatus* and carrying pollen grains (Fig. 1C,D). We presumed that the birds visited the flowers for nectar, which they obtained mostly while perching, although hovering was also recorded (Fig. 1C,D, Supplementary Data - Video). Obvious yellow spots of pollen on their heads were usually observed after they had visited a flower (Fig. 1A,C). The flocks moved swiftly from one clump of *A. acuminatus* to another; these were generally *c*. 10–50 m apart. Each bird actively visited 3–4 flowers per clump, resulting in almost all the flowers having been visited after a flock had passed through. We found that all the individuals at the site were visited by these passerine flocks within a day. The birds were also seen frequently visiting flowers of other nearby species, including *Prunus campanulata* (Rosaceae, trees up to 10 m high growing along the roadsides) and *Machilus thunbergii* (Lauraceae, large trees with tiny flowers blooming in the canopy.)

No other pollinators or visitors to *A. acuminatus* were recorded in our observations or video recordings. Insect activity, including hawk moths, in the understory appeared to be low during the flowering season of *A. acuminatus* (November to February). Furthermore, no bats were seen at the site during the study period.

### Morphological comparison

**Figure 2:**
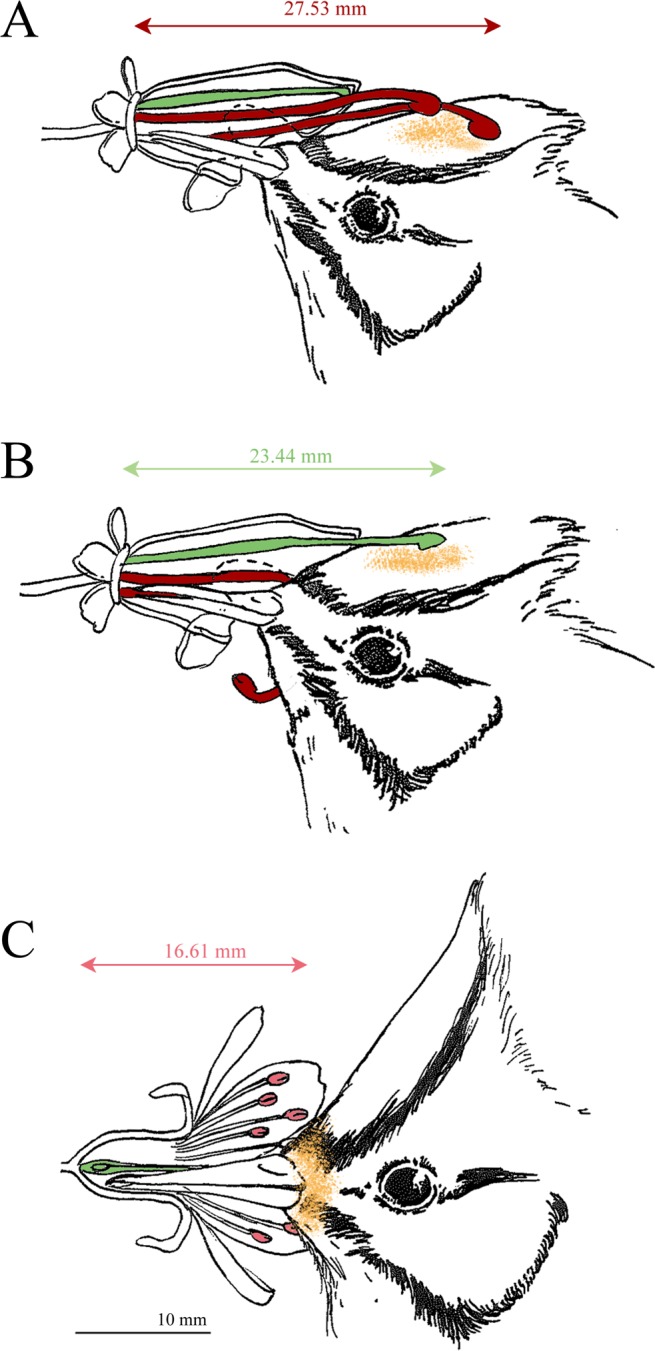
Comparison of floral structures between the male and female stages of *A. acuminatus* and a co-flowering species, showing a visiting Taiwan Yuhina (*Y. brunneiceps*). The stamens of *A. acuminatus* are colored dark red, the anthers of *P. campanulata* are light pink, and the pistils of both species are indicated in green. Yellow dots indicate the potential pollen placement on the bird, as estimated from morphological measurements. The key characters in the illustrations are all drawn according to the same scale, shown at the bottom of the figure. (**A**) Male stage of *A. acuminatus*. The protruding stamens in this phase deposit pollen on the Yuhina’s crest. (**B**) Female stage of *A. acuminatus*. The extended gynoecium reaches almost exactly the same point on the Yuhina’s crest. (**C**) Co-flowering species, *P. campanulata*. Both the stamens and the gynoecium reach the base of the Yuhina’s beak.

Fresh *A. acuminatus* flowers (n = 24) had an average corolla width of 5.8 ± 0.16 mm. The dorsal and ventral corolla tube lengths were 17.5 ± 0.26 and 7.9 ± 0.21 mm, respectively. In male-phase flowers (Fig. 2A), the first pair of stamens was 28.2 ± 0.32 mm long, and the second pair 24.2 ± 0.27 mm. The pistil inside the corolla tube was 18.2 ± 0.98 mm long (n = 13). When entering the female phase (Fig. 2B), the filaments of both pairs of stamens had already curled downwards. In this phase, the pistil extended outwards and was 23.4 ± 2.05 mm long (n = 9).

Similarly, the sepals of *P. campanulata* form calyx tubes, where generalist passerines can probe for nectar (Fig. 2C). The floral tube of *P. campanulata* had an average calyx width of 4.7 ± 0.16 mm and length of 8.6 ± 0.12 mm (n = 18). The stamens of *P. campanulata* were in clusters of two different lengths, and the longer ones extended 16.6 ± 0.29 mm from the bottom of the calyx tube (n = 18). The pistil extended almost as far as the outer layer of stamens.

The primary pollinator of *A. acuminatus*, the Taiwan Yuhina, had an average head length (from the back of the skull to the base of the bill) of 29.6 ± 0.09 mm (n = 119). Its bill, which probes into flowers for nectar, was 11.7 ± 0.04 mm long and 4.5 ± 0.03 mm wide (n = 119). Its crest, an iconic feature of this species, was 26.9 ± 0.12 mm long and 9.0 ± 0.08 mm wide (n = 119).

The length of the stamens during the male phase and the elongated gynoecium of *A. acuminatus* approximately match the length of the bill and half of the crest of the Taiwan Yuhina (Fig. 2A,B). In contrast, the stamens of *P. campanulata* are just slightly longer than the Yuhina’s bill. The anthers of these flowers would therefore touch the bird around the base of its bill during visitation (Fig. 2C). The Yuhina’s bill is narrower than both the *P. campanulata* calyx tube and the *A. acuminatus* corolla, allowing adequate space for the bird to probe for nectar without damaging the flowers.

### Pollination effectiveness

Natural fruit set was 60% (estimated from 27 randomly selected inflorescences). In each inflorescence, an average of 2.4 ± 0.22 fruits was recorded, from an average of 4.0 ± 0.04 flowers.

**Figure 3:**
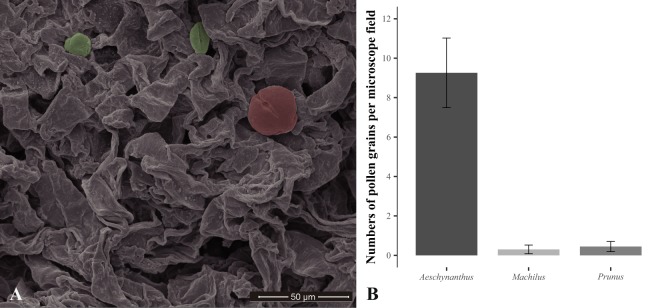
Pollination effectiveness in terms of conspecific pollen transfer. (**A**) A microscope field from a 1500× SEM image of an *A. acuminatus* stigma. A pollen grain from *Prunus campanulata* is colored pink, while two typical pollen grains from *A. acuminatus* are shown in green. (**B**) The average number of pollen grains in a microscope field. Error bars indicate the standard error of the mean.

We counted a total of 1615 pollen grains from 15 stigma samples. Each stigma was counted separately with SEM, and at least two non-overlapping *c*. 34,000 μm2 fields (1500×) were chosen for each stigma, for replication. *Aeschynanthus acuminatus* has small (*c*. 15 μm in diameter), prolate pollen grains (Fig. 3A). These were easily distinguished from the next-most-common pollen, that of *P. campanulata*, by size (*c*. 25 μm in diameter) and shape (near spherical). There were on average 9.3 ± 1.76 *A. acuminatus* pollen grains in a microscope field, representing 94% of the total pollen count. Heterospecific pollen grains were from *P. campanulata* and *M. thunbergii*, with 0.4 ± 0.26 and 0.3 ± 0.22 grains in a microscope field, respectively (representing 6% and 0.6% of the total pollen count) (Fig. 3B).

### Pollination experiments

We have conducted pollination treatments counting fruit sets from artificial selfing and crossing flowers, together with bagged non-treated flowers (a test for autonomous selfing) and naturally pollinated flowers of *A. acuminatus* (Table 1). The fruit sets from the naturally pollinated flowers received the highest percentage (60%) following by that from the artificial crossed flowers (40%). Artificially selfed flowers (geitonogamy) also generated fruit set but the proportion is low (18%). Bagged flowers did not receive fruit set thus autonomous selfing may not be possible. The reason why the fruit set of artificial crossed flowers is lower to that of naturally pollinated flowers may be due to the limited sample size in artificial crossing treatment.

**Table 1:** Fruits sets from pollination treatments.

	Natural control	Bagged	Artificial selfed	Artificial crossed
Fruit set (%)	60	0	18	40
Sample size (Flowers/Inflorescences)	108/27	7/7	11/11	15/15

### Nectar production and properties

Copious nectar production was measured in bagged flowers (60.5 ± 12.93 µL). The average nectar volume at 10:30 (71 ± 16.84 µL) was higher than that recorded at 16:30 (51.8 ± 19.76 µL), although this difference was not significant (t = 0.74, *p* > 0.05). The concentration of bagged flowers was constantly low through time (6.3 ± 0.69%; t = 0.72, *p* > 0.05).

**Figure 4:**
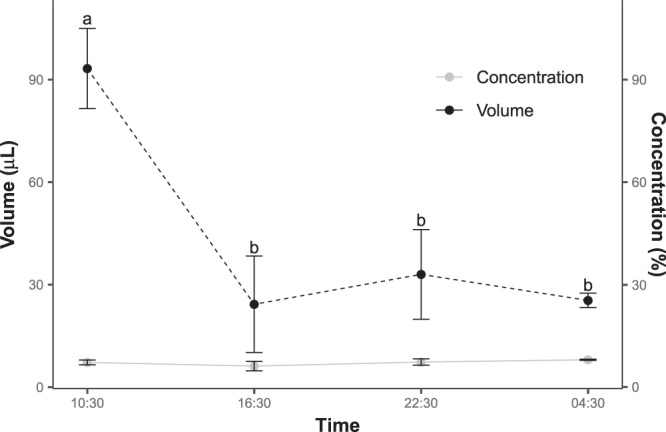
Changes in nectar concentrations and volume of unbagged flowers (standing crop) through time. The concentration did not change significantly throughout the day but the standing crop dropped dramatically during the daytime (10:30 to 16:30). Statistically homogeneous groups with respect to nectar volume are indicated by letters (a,b) above the error bars. Error bars indicate the standard error of the mean.

The standing crops, which were measured from unbagged flowers exposed to pollinators, also exhibited a peak at 10:30 (Fig. 4). The average nectar volume increased from 25.4 ± 2.12 µL at 04:30 to 93.3 ± 11.74 µL at 10:30, and then decreased to 24.3 ± 14.12 µL at 16:30 and 33 ± 13.13 µL at 22:30. A one-way ANOVA revealed a significant difference between the standing crops of nectar at different times of the day (*F* = 8.52, *p* < 0.01). In particular, the nectar volume at 10:30 was significantly higher than at all other times (post-hoc comparisons using Tukey’s HSD test; Supplementary Data Table S1). The significant decline in the quantity of standing crop from 10:30 to 16:30 was congruent with the observations of pollinators visiting during the late morning. The increase in standing crop between 05:00 and 10:30 suggests that nectar recovery via secretion occurs during early to mid-morning.

The average nectar sugar concentration of standing crops was low (7.2 ± 0.44%), with no significant differences among the times of the day (*F* = 0.70, *p* > 0.05; Fig. 4). The nectar sugar was composed of fructose (49%) and glucose (51%), with no detectable sucrose. There were no differences in nectar sugar composition (hexose percentage) among floral stages or time periods.

### Floral reflectance spectra

**Figure 5:**
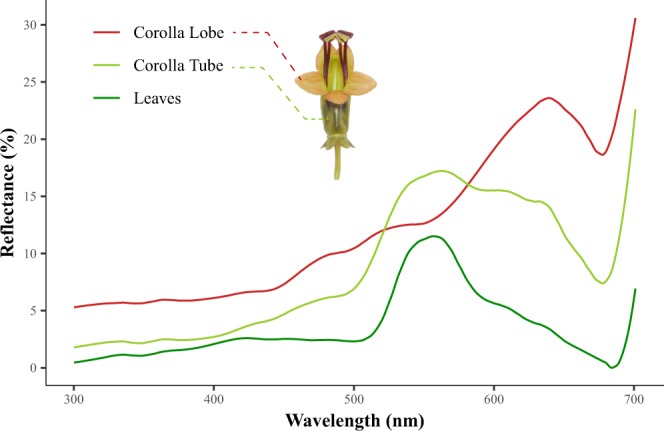
The reflectance spectra of two floral parts and the leaves of *A. acuminatus*. The substantial reflectance in the long-wavelength range (450–700 nm) of the corolla lobe resembles the typical pattern of bird-pollinated red flowers. The reflectance pattern of the greenish corolla tube resembles that of the leaf, which is typical of green plant tissues.

The spectral reflectance of corolla lobes was minimal from 300–450 nm, but increased between 450–700 nm, with a peak at 640 nm and a trough at 680 nm (Fig. 5). The corolla tubes showed notable but decreasing spectral reflectance at 540–640 nm, and a trough at 680 nm the red color region. The spectral reflectance of the leaves was typical of that of the green vegetative parts of all plants, with a major peak at 550 nm and an increase in reflectance from 680 nm upward. Thus, the reflectance spectra of the corolla tubes and leaves for the range 300–680 nm are relatively similar, with the highest peak around 550 nm, whereas the highest peak of the opening side of corolla lobes is at 640 nm. Thus the facing view of the corolla lobe are in typical red color reflectance but the corolla tube color has shifted to green, and a similar spectrum between corolla tube and foliage leaves was found.

## Discussion

### Generalist passerines as exclusive and effective pollinators

We found that generalist passerines were exclusive and effective pollinators of *A. acuminatus* in Taiwan, subtropical East Asia—a sunbird-absent region that has long been thought to lack bird-pollinated plant species^3,15^. In this study, three passerine species, the Taiwan Yuhina, the Grey-cheeked Fulvetta, and the White-eared Sibia, were recorded as effective pollinators visiting winter-blooming *A. acuminatus* in understory habitats. In contrast to *Camellia japonica* and *Eriobotrya japonica*, which adopt mixed pollination systems comprising both birds and insects^14,17^, *A. acuminatus* appears to be exclusively pollinated by generalist passerines, based on its flowering time and floral configuration. The low temperatures and heavy moisture in the forest understory limit insect activity during the winter, and we only observed insects visiting flowers of other species frequently outside the forest. In addition, the corolla tube of *A. acuminatus* is dorsally extended, lacking a landing platform on its ventral side. This would make it difficult for most insect visitors to access the nectar, but not for birds, bats, or hawk moths. None of our video recordings or human observations recorded hawk moths or bats visiting *A. acuminatus*, so it is exclusively pollinated by generalist passerines. Together with the data that the natural fruit set is high (60%) and mainly conspecific pollen (ca. 100%) was found on the stigma of *A. acuminatus* after passerine visiting, in comparison to that of the two other reported cases of generalist passerines pollinating plants in East Asia (36.2% in *C. japonica*^14^ and 2.6% in *E. japonica*^17^). We thus believe that the pollination effectiveness by generalist passerines on *A. acuminatus* is remarkably high, although further verification on fruit set difference between natural pollination and caged flowers, in which birds are excluded, is needed.

### Winter-blossoming *A. acuminatus* synchronizes with the seasonal diet of generalist passerines

Several reports have demonstrated that plants pollinated by generalist passerines in East Asia are winter flowering in synchronizing with the food shortage season of generalist passerines^3,14,17,22^. Nectarivorous feeding behavior is rather uncommon in passerines except sunbirds and white-eyes but our finding together with previous reports confirmed Grey-cheeked Fulvetta (*Alcippe morrisonia*), Taiwan Yuhina (*Yuhina brunneiceps*), and White-eared Sibia (*Heterophasia auricularis*) actively visits *A. acuminatus* and some winter flowering species for food^31,32^. They undergo a seasonally transitional diet for nectar during the winter, even though they feed primarily on fruits and insects in other seasons^33,34^. Chen and Chou^31^ found that the nectar of winter-blooming *A. acuminatus* accounts for 78% of the food intake in Taiwan Yuhina when they migrate to lower altitude in the food-scarce winter. The other passerine pollinator, the Grey-cheeked Fulvetta, inhabiting the low-altitude subtropical forest all year round, also switches from an omnivorous to a nectarivorous diet in winter (November to February)^31^. In summary, these two generalist passerines exhibit a seasonal diet on nectar during the winter, similar to the strong specialization of the Japanese white-eye’s diet to the nectar of *C. japonica*^14^.

### Morphological adaptation between *A. acuminatus* and generalist passerines

Birds with morphological, physiological, and behavioral differences select different floral characteristics in the plants they pollinate^35^. Generalist passerines are usually larger in body size than specialist birds, with shorter but wider bills^15^. In accordance with these characteristics, most of the flowers pollinated by generalist passerines have relatively open corolla tubes with protruding anthers allowing the short-beak passerines to approach the nectars^36^. The relatively wide corolla tubes of *A. acuminatus*, compared with the long, narrow corolla tubes of congeneric species, allow for probing by the thicker bills of passerine birds (Fig. 2A,B), and indicate an obvious key character of adaptation.

Many nectarivorous birds including sunbird, hummingbird and honeyeaters have specialized tongue morphologies to facilitate nectar sucking^37^. In certain species of generalist passerines, such as the omnivorous white-eyes (Zosteropidae), have brush-tipped tongues to facilitate nectar sucking, as in honeyeaters^15^. However, the Taiwan Yuhina and White-eared Sibia have also evolved brush-tipped tongues specialized to quickly collect nectar via capillarity^32^. Regarding their flower visiting behavior, although generalist passerines have been reported to visit flowers predominantly by perching, our study found that the Yuhina sometimes visited flowers by hovering (Fig. 1D), which is considered an adaptive behavior of specialized nectarivores such as hummingbirds and some sunbirds^38,39^. Together with the seasonal winter dietary specialization, the presence of hovering behavior and the brush-tipped tongue suggest that these two generalist passerines may have established a stable specialized relationship with *A. acuminatus*, contrary to the expectation that generalist passerines are only loosely associated with nectar-providing plants.

### Highly efficient pollination by generalist passerines provides reproductive success

Passerines have been reported to forage and travel in groups and can be effective in cross-pollinating^40^. We also observed that the generalist passerines always visited the clumps of *A. acuminatus* in mixed flocks of two to four species numbering about 20 individuals. For White-eared Sibia, they moved on to the next clump until almost all the flowers in a clump had been visited. For Taiwan Yuhina, they tended to visit just few couple of flowers of each plant and quickly moved to next plant. As a result, each Yuhina individual spent only a short time on a few flowers in a clump. This type of feeding behavior would provide *A. acuminatus* with a high chance of outcrossing and a large pollen dispersal distance, as has been hypothesized for typical bird-pollinated species in other studies^5,41,42^.

Exact pollen deposition on the pollinator body and the precise delivery of pollens onto the stigma of the flower are important for the reproductive success of flowers^43^, and provides an explanation for high pollination effectiveness of *A. acuminatus*. We found the generalist passerines also frequently visited the flowers of both *M. thunbergii* (tiny) and *P. campanulata* (wide open, with a shorter actinomorphic corolla) for nectar. Presumably, *A. acuminatus* could potentially suffer from pollen interference from these sympatric co-flowering species that share the same pollinators. Floral morphology comparison, however, illustrated that the pollen placements of *A. acuminatus* and co-flowering species are on different body parts of the generalist passerine pollinators to minimize pollen interference. Patches of pollens of *A. acuminatus* could be seen on the crests of the Taiwan Yuhina (Fig. 1A), while pollens of *P. campanulata* are at the base of the bird’s bill (Fig. 2).

Heterospecific pollens on the stigma can significantly reduce the fitness of a species due to stigma or style clogging, reduced seed set, and unfit hybrid offspring^6,44^. The strongly differential pollen placement on the body of Taiwan Yuhina appears to be the key to the almost 100% conspecific pollen load on the stigmas of *A. acuminatus*. From an ecological perspective, this mechanism allows a set of co-flowering species to coexist, even relying on the same pollinator, retaining diversity within the community^43,45,46^.

### Effective pollination by generalist passerines inferred from high natural fruit sets

Our pollination experiment results demonstrated that the fruits set from the natural pollination (60%) and artificial crossing (40%) achieved much higher success than artificial selfing (geitonogamy) (18%) (Table 1). Successfully fruit sets from artificial selfing revealed self-compatibility, but cross-pollinated flowers produced greater fruit sets than self-pollinated, implying outcrossing is the major reproductive strategy of *A. acuminatus*. Although successful fruit sets can be observed in artificial selfing, it may not contribute to the naturally high fruit-set because the stamen and pistil are herkogamous when mature (Fig. 1B) and autonomous selfing is impossible – fruit set from bagged flowers is 0% (Table 1). In addition, the flowers of *A. acuminatus* are protandrous^26,27^, in which anthers shed pollens before the stigma become receptive (Fig. 2A,B). Even though *A. acuminatus* is self-compatible, due to herkogamy, any successful natural fruit set must rely on cross-pollination by pollinators. This is supported by our observations of the feeding behavior of the generalist passerines particularly the Taiwan Yuhina: one individual usually spent a short time on only a few flowers per individual plant, indicating a general likelihood of outcrossing in *A. acuminatus*.

The naturally high fruit-set indicated the effectiveness of generalist passerines as pollinators for the reproductive success of *A. acuminatus*. When comparing the resulting fruits set of *A. acuminatus* to other passerine-pollinated winter flowering species, the average 60% natural fruit set is at a similar high level as that (75–80%) in Yuhina-pollinated *B. hancei* (Scrophulariaceae)^22^ but is much higher than that (20–40%) of *Zosterops* (Japanese white-eyes)-pollinated *C. japonica*^14^. And the fruits set of passerine-pollinated *A. acuminatus*, *B. hancei* and *C. japonica* are even higher than that (20–30%) of sunbird-pollinated *C. petelotii*, suggesting generalist passerine may act as an effective pollinator in the sunbird-absent regions of East Asia^14,23^.

### Low sugar concentration and hexose dominant diluted nectar fits the generalized bird pollination system

From the aspect of reward, flowers pollinated by birds usually secrete vast quantities of nectar in diluted concentrations^11,47^. Studies on the nectar properties of bird-pollinated species in East Asia were extremely limited until recently, when it was reported that several bird-pollinated species release large amounts of diluted nectar^17,22,48,49^. Our results regarding the large quantity (61 µL) and low sugar concentration (7%) of nectar produced by *A. acuminatus* support similar conclusions from these studies.

In a recent survey over 2100 bird-pollinating species, it was found that high nectar sugar concentration is associated with specialist bird pollinated flowers and low nectar sugar concentration for generalist bird pollinated plants^50^. Within bird-pollinated flowers, specialist nectarivorous birds such as hummingbird and honeyeater favor flowers with higher concentration of nectar (average in 23.2 vs. 21.6%, respectively), while passerine-pollinated flowers produce copious and very dilute nectar (10–15%)^51,52^. As to the nectar sugar composition, hexose-dominant nectars have been predicted to be associated with the generalized bird pollination (GBP) system (i.e. plants with a wide range of opportunistic birds as pollinators), while sucrose-dominant nectars evolved with the specialized bird pollination system^12,13,53^. This was explained that specialist birds can absorb sucrose, but opportunistic nectar feeders such as generalist passerines can only digest hexoses^12^.

The hexose-dominant nectar of *A. acuminatus*, composed of fructose (49%) and glucose (51%) and no detectable sucrose, supports our contention regarding this as a generalist passerine pollination syndrome. Similarly, flowers in East Asia belong to GBP system such as in *B. hancei*, *E. japonica* and *Rhodoleia championii* have been characterized by large volumes (*c*. 40–100 µL) of extremely diluted nectar (*c*. 9–12% w/w)^17,22,48^, relative to those pollinated by specialized nectarivores (i.e., sunbirds and hummingbirds), which produce medium volumes (*c*. 10–30 µL) of moderately diluted nectar (*c*. 15–25% w/w). The hexose-dominant nectar of *A. acuminatus* is therefore toning with the predicted “nectar generalists” in East Asia.

In Gesneriaceae, the focus of pollination biology and nectar quantity analyses has mainly been on New World lineages^54–57^. A pioneering study by Perret *et al*.^54^ revealed that the nectar of hummingbird-pollinated *Sinningia* species has a high sugar concentration (23.9 ± 10.6%) and is sucrose dominant (*c*. 57–86%). The reason why these ornithophilous *Sinningia* species have sucrose dominant high sugar nectar is probably because they were phylogenetically derived from bee-pollinated ancestors (28.7 ± 10.6% sugar concentration). The nectar sugar properties of *A. acuminatus* and few sampled Southeast Asian *Aeschynanthus* species^25^, on the other hand, are low in sugar concentration (7%) and rich in hexose (45.6% fructose and 53.3% glucose) containing only traces of sucrose (0–3.6%), fit better with GBP (see above) rather than specialist bird pollination system. Although *Aeschynanthus* species in SE Asia have long been proposed by sunbird pollination^25–27^, the nectar sugar composition data may imply that *Aeschynanthus* species in sunbird-rich Southeast Asia are actually pollinated by generalist passerines, instead of the long-suspected sunbirds. An alternative explanation is that *Aeschynanthus*-visiting sunbirds may have already adopted to this low nectar sugar composition. Further evidence from thorough and extensive field observations on pollinators for other *Aeschynanthus* species and nectar analyses are needed to discriminate these hypotheses.

### Reflectance spectrum of corolla lobe revealed a major peak in long-wavelength red but shifting to green in corolla tube

In terms of the visual attraction of flowers, typical bird-pollinated flowers have their major reflectance peaks in the long-wavelength yellow to red spectrum (600–700 nm), which is thought to exempt insects, especially bees, from visiting flowers, but not birds, which have a greater hue discrimination ability^3,8,9,58,59^. The facing view of the corolla lobe of *A. acuminatu*s reflects typically red color at a peak at 640 nm (Fig. 5). However, the floral reflectance of the corolla tube has extended its reflectance spectrum to a peak at 550 nm, in a similar pattern as the reflectance of the leaves. The extension of reflectance to 550 nm is evident from the relatively green color of the corolla tube, in contrast to the reddish color (major peak at 650 nm) on the opening side of the corolla lobe (Fig. 5). The flower with green corolla tube and reddish lobe in *A. acuminatu*s is therefore dramatically different from all other sunbird-pollinated Southeast Asian *Aeschynanthus* species in which their corolla color are vivid red^27^. Nonetheless, *A. acuminatu*s still keeps its pollinator facing side of the corolla lobe, inner corolla tube and stamen in red contrasting to outer corolla tube in green generating a red-green contrast perhaps to attract birds (Fig. 1). Based on the phylogeny of *Aeschynanthus*^28^, the green flower color in *A. acuminatus* is a derived trait from other red colored putatively sunbird-pollinated *Aeschynanthus* species. The reason why the color of corolla tube in passerine-pollinating *A. acuminatus* have shifted from red to green is not clear but the possible explanation is given below.

Bird pollination plants may have evolved red color to deter ineffective pollinators such as bees and other insects^8,9^. Bees have only a limited ability to distinguish red flowers because most bees have their longest wavelength ‘green’ receptor with the sensitivity peak up to 540 nm which make them difficult to discriminate red flowers from background green foliage^8,9^. Bees are thus less effective in pollinating red flowers than birds because they require more searching time to identify red flowers^7,9,58,59^. One explanation that why flower color of *A. acuminatus* is green could be contribute to its phenology. The flowering time of *A. acuminatus* have shifted to winter when there is very low insect activity. It is therefore no need to evolve red color to deter bees and other insects in this cold weather condition. In situations of bee–bird competition, birds will impose a selection force on those flowers to evolve towards redder colors, which then become more specialized for pollination by birds^9^. In the absence of bees, the selective pressure on floral red color could therefore be released, allowing *A. acuminatus* flower to lose its ancestral red color and become relatively greenish. In fact, the floral colors of *A. acuminatus* among its populations (different localities within Taiwan, and also Vietnam and China) demonstrates a pattern of gradation between completely green and red (personal observation). This variation might act as an evidence of relaxation from selection on red color.

### Adjusted pollination syndromes for pollinator shifts in peripheral distribution and successful colonization

When a plant species extend beyond the distribution of its major pollinator, shifts to new local effective pollinators is necessary to ensure its reproductive success^60,61^. Given the wide distribution of *A. acuminatus*, from northern Thailand to southeastern China, mainly beyond the northern limit of sunbird distribution, a shift to differential pollinator assemblages among different populations could be expected. The shift of *A. acuminatus* to generalist passerines pollination appear to have played a crucial role to facilitate its successful colonization to Taiwan, one of the northernmost extensions of the range of *Aeschynanthus*. Because the peripheral distribution of *A. acuminatus* from the range of all other *Aeschynanthus* species, the divergence of floral traits could occur rapidly caused by the limited gene flow to congeneric species at the distribution center and the selection on pollination syndromes from local effective pollinators. The distinct floral morphology and bird pollination syndromes evolved in *A. acuminatus* supports this hypothesis. The evidences include: the wide-open corolla tube allowing short-beak passerines to approach the nectars, winter-flowering phenology to synchronize with the food shortage season of passerines, exact pollen deposition on the crests of Taiwan Yuhina with high percentage of conspecific pollen placement on the stigma, low sugar concentration but hexose-dominance nectar composition in favour of generalized bird pollination, and corolla lobe with stamen in red but outer corolla tube shifting to green for visual contrast. On the other hand, the evolved brush-tipped tongue for nectar sucking in these generalist passerines and nectars as their major diet in winter also suggest that these birds have established a seasonally specialized relationship with *A. acuminatus*. The resulting effective pollination by matching these floral traits to passerine’s behaviour likely to help *A. acuminatus* to colonize island habitat such as Taiwan. Further investigation of population genetic structures and the pollinator assemblages of other populations across the entire range of *A. acuminatus* in East Asia will shed light on the temporal pattern of pollinator shifts and how these generalist-passerine pollination syndromes were evolved in such an effective way.

This is the first reported case of a specialized relationship between generalist passerines and an ornithophilous plant species in sunbird-absent Taiwan, the subtropical island of East Asia, a region generally lacking bird pollination record. The successful adaptation of *A. acuminatus* to sunbird-lacking habitats suggests that the role of generalist passerines in plant–pollinator interactions and floral evolution in the sunbird-absent regions of East Asia may be underestimated.

## Methods

### Study sites

Our study was conducted at two sites in the broadleaf forests of Sanxia Township in Taiwan. Pollinator observations were conducted at Qingshui Bridge [QSB, 24°50′22.0″N, 121°26′56.0″E, *c*. 340 m above sea level (ASL)], New Taipei City, on seven clusters of individuals of *A. acuminatus*, and Xitou Nature Education Area (XT, 23°40′28.3″N, 120°47′42.2″E, *c*. 1100 m ASL), Nantou County, throughout the park. Quantification of pollination syndromes (nectar properties and floral reflectance) was mainly operated at QSB. Experiments for testing breeding systems and the percent fruit set (i.e., how many flowers resulted in fruit set) were mainly conducted in the Manyueyuan National Recreation Forest Area (MYY, 24°49′33.0″N 121°26′54.0″E, *c*. 400 m ASL), New Taipei City. Individuals of *A. acuminatus* occur on almost every tree along the trails throughout the forest of XT and MYY. The study sites are characterized by a subtropical monsoon climate of a cold humid winter with average temperature of 14.7 °C, a lowest temperature of 4 °C, and relative humidity of 87% from December to February at MYY and QSB (data from Manyueyuan National Forest Recreation Areas) and average temperature of 12.7 °C from December to February at XT (data from Xitou Nature Education Area).

### Pollinator observations

Characterization of the pollinator fauna was conducted in February and March 2016 at QSB and in December 2018 at XT. Floral visitors were continuously recorded throughout the day, for 16 hours by human observers and for 48 hours by video cameras. Human observations were conducted in 30-min censuses per 2 hours in the day and 15-min censuses per 4 hours at night at QSB on several non-consecutive days and in 30-min censuses per hour in the day at XT. Nocturnal observations were also conducted at QSB by both human observers and video cameras, to avoid expectancy bias. Video cameras with infrared night vision (Sony Handycam series) were used for nocturnal observations. Floral visitors were identified to the species level. The time and duration of floral visits, behaviors during visitation, and types of floral resources collected were recorded.

### Morphological comparison

To reconstruct and compare the processes of pollen transfer of three plant species (*A. acuminatus* and two co-flowering species) by generalist passerines, fresh flowers from both study sites QSB and MYY were collected and their characteristics were quantified. Morphological characters related to pollination processes, namely corolla length and width, and stamen and gynoecium lengths (or analogous characters in different species), were defined and measured. Flowers of *A. acuminatus* are dichogamous^26,27^ (protandrous). Therefore, we arbitrarily categorized each flower into either a male or a female phase, based on its morphology. Samples comprising 15 male-stage and nine female-stage *A. acuminatus* flowers, and 18 individual flowers of the nearby bird-pollinated *Prunus campanulata* were used in this comparison.

The morphological measurements of one of the bird species observed to be a flower visitor, the Taiwan Yuhina (*Yuhina brunneiceps*), were provided by Dr Sheng-Fen Shen from the Biodiversity Research Center of Academia Sinica, Taiwan. The facial characters related to probing behaviors—bill length and crest length—of 115 individuals measured in 2007 were averaged and used in this comparison.

The actual fitting mechanisms may be affected by the length of tongues, however, none of the nectar feeding behaviors involved with tongues has been recorded. The previous study^32^ demonstrated the tongues of Taiwan Yuhina is 15.4 mm, which indicates at most 4 mm differences when tongue exceeds beak for feeding. Beaks can act as a constant proxy can well support our comparisons of relative pollen placements of co-flowering species and mechanical fit (beak width) of congeneric species, especially when feeding modes are unknown.

### Pollination effectiveness

To evaluate the pollination effectiveness of generalist passerines in *A. acuminatus*, natural fruit set and stigma pollen-load composition were recorded. Each inflorescence always contains four sequentially blooming flowers. Fruit set was estimated by counting the number of elongated capsules on randomly selected inflorescences (n = 27) after the flowering season at the study site MYY.

Stigma pollen loads were observed under a scanning electron microscope (SEM) to determine the effectiveness of conspecific pollen transfer. Accessible fresh female flowers were randomly collected from 7 individuals with at least 20 m distance from each other along the trail in MMY (average 1–3 flowers per individual). To allow sufficient exposure time to pollinators, all stigmas from flowers in the female stage were collected in the late afternoon. The flowers on their first day in transition into female stages were also avoided. The stigmas were then sliced by scalpel and coated with gold particles for observation. Up to 20 photographs of each stigma were taken under 1500 × magnification for pollen identification. Pollen depositions were identified via pollen morphology to the species level, according to pollen references of co-flowering species at the study site. Total pollen loads were counted twice and averaged to obtain the percentage of conspecific pollen transfer.

### Pollination experiments

Pollination experiments conducted in the field were designed to investigate the contribution of pollinators (fruit set from natural control) and possible breeding strategy (selfing or outcrossing) of *A. acuminatus*. For natural control treatment, we counted samples of 108 flowers from 27 inflorescences of naturally grown individuals at QSB site, the mountain area in New Taipei City. Because the fruit set from natural individuals could possibly be resulted from pollinator visiting and/or autonomous selfing, we also conducted artificial selfing (geitonogamous self-pollination between different flowers of the same individuals) and crossing (xenogamous cross-pollination between different individuals) from hand pollinations. Bagged flowers (without any pollination treatment) since pre-anthesis throughout the fruiting were set up to evaluate the possibility of autonomous selfing.

### Nectar production and properties

To determine the secretion pattern of nectar over a 24-hour period, nectar volume was measured from 15 bagged flowers (bagged with polyester non-woven bags before they opened) on five individuals from the study site QSB at 10:00 and 16:30. To quantify the standing crop of nectar in a daily cycle for evaluating the change in nectar quantity with pollinator visitation, the nectar volume of 25 unbagged flowers on five individuals from the study site QSB was also measured at 04:30, 10:30, 16:30, and 22:30. The nectar volume of each flower was measured only once, using a 20-µL capillary tube to extract all the nectar.

Next, 20 µL of nectar from each sample collected at the study site QSB was used to determine the nectar concentration (grams of sucrose equivalents per 100 g of solution) with a MASTER-T handheld refractometer with the range of 0–33% (ATAGO, Tokyo, Japan) in the field. The remainder of the nectar samples were stored on ice, then stored at −80 °C until the sugar composition analysis was conducted.

The nectar sugar constituents (glucose, fructose, and sucrose) were separated and quantified using high-pH anion-exchange chromatography (HPAEC) combined with pulsed electrochemical-detection (PED) on a P200 Spectra-Physics chromatography system (Spectra-Physics, Fremont, CA, USA). The sugars were separated in a Dionex CarboPac PA10 column (Thermo Scientific, Waltham, MA, USA) with 160 mM NaOH as the elution buffer. Calibration was conducted with a standard solution of each of the sugars. The nectar sugar composition of six nectar samples was determined, and the average was taken.

### Floral reflectance

We used an Ocean Optics USB4000 spectrophotometer and a fiber optic reflection probe (UV/VIS 400 micron; Ocean Optics, Largo, FL, USA) to objectively measure the reflectance spectra of the corolla tubes, lobes, sepals, and green leaves of *A. acuminatus* at the study site QSB. The probe was held at a 5-mm distance and a 45° angle to the sample’s surface. An Ocean Optics DT-mini deuterium tungsten halogen light source with a 200–1100-nm spectral range was used. Calibration was conducted with an Ocean Optics WS-1 diffuse reflectance standard.

## Supplementary information

Dataset 1

Supplement Video

## Data Availability

All statistics are presented in the main text. Raw data and R codes for data analyses and visualization are available on Github repository: https://github.com/jingyilu/Pollination-of-Aeschynanthus-acuminatus.
