# Supplementary material for: Effective pollination of Aeschynanthus acuminatus (Gesneriaceae) by generalist passerines, in sunbird-absent East Asia
Source: Sci Rep. 2019 Nov 26;9:17552. doi: 10.1038/s41598-019-53035-2 (PMC6879542; doi:10.1038/s41598-019-53035-2)
Supplement: Supplementary file 1 — Dataset 1 [file 41598_2019_53035_MOESM1_ESM.pdf]

Supplementary Information

Effective pollination of *Aeschynanthus acuminatus* (Gesneriaceae) by babblers, generalist passerines, in sunbird-absent East Asia

Kai-Hsiu Chen[1†] , Jing-Yi Lu[1†], Chun-Neng Wang[1,2*]

[1]Department of Life Science, National Taiwan University, Taipei, 10617, Taiwan

[2]Institute of Ecology and Evolutionary Biology, National Taiwan University, Taipei, 10617, Taiwan

*Correspondence author (email: leafy@ntu.edu.tw)

[†]These authors contributed equally to the work.

Table S1. Post-hoc analysis of changes in nectar volumes through the day (Tukey's HSD test).

**Pair Comparisons**	**Differences**	**Lower**†	**Upper**†	**Adjusted *p*-value**
**1630–1030**	−69.000	−116.59969	−21.40031	**0.0048916****
**2230–1030**	−60.250	−107.84969	−12.65031	**0.0125986***
**0530–1030**	−67.875	−115.47469	−20.27531	**0.0055178****
**2230–1630**	8.750	−38.84969	56.34969	0.9459314
**0530–1630**	1.125	−46.47469	48.72469	0.9998686
**0530–2230**	−7.625	−55.22469	39.97469	0.9630350

†Lower and upper boundaries of the 95% confidence intervals of the differences.
* $p < 0.05$, ** $p < 0.01$.
